# Soft Robotics

**DOI:** 10.3390/biomimetics4010022

**Published:** 2019-03-12

**Authors:** Barbara Mazzolai

**Affiliations:** Center for Micro-BioRobotics, Istituto Italiano di Tecnologia, 56025 Pontedera, Italy; barbara.mazzolai@iit.it

## 1. Introduction

In Nature, the adaptability of many organisms and their capability to survive in challenging and dynamically changing environments are closely linked to their characteristics and the morphology of their body parts. Mechanical interactions are used to more effectively process information and to have a more rapid response of movements and actions.

Soft, adaptable, compliant and variable-stiffness components, which are typical of natural body parts, are the key elements of this mechanism. Their particular structure and their specific biomechanical behavior intrinsically facilitate the physical contact, dynamic adaptability and consequent response. This aspect is particularly relevant to robotics, where we look for new strategies to provide robots with more advanced and efficient abilities to operate rapidly and robustly in a variety of environments, and to be safer during interactions with humans.

Soft robotics is the new trend in robotics that is exploring this route by designing and fabricating adaptable, smart, variable-stiffness materials, sensors and actuators, and by using their morphology and biomechanical characteristics for new dynamic behaviors.

This Special Issue of *Biomimetics* gathers research articles and reviews with technological studies and trends in soft robotics. Advancements in artificial soft sensors, new fabrication methods for self-sensing actuators, hand exoskeletons for manipulation, new devices and strategies for the mechanical reinforcement of heart muscle walls, new ways to develop smart continuum robots, and novel methodologies for the definition of control architectures for complex behaviors, are major topics of this collection. 

The research arguments focus their attention and drive the development of technologies for systems dealing with unstructured environments and interacting with humans, where the soft robotics approach may help go beyond capacities of current robotics systems.

## 2. Sensory Systems for Perception and Actions

Sensory organs for mechanical sensing are of paramount importance for reducing the computational load and elaborating the information gathered during mechanical interactions, as well as performing movements and actions. They use biomechanics to pre-process the sensory information via morphological computation and thus partially compensate the activity of the neural part. 

The review paper by Astreinidi Blandin et al. [1] focuses on biomechanical aspects of sensing structures in some animals and plants, such as their structure and materials. The analysis of mechanical aspects includes the orientation of the sensory element, the shape, the distribution, the material properties, and the micromechanics. In this way, the work covers a range of sensory organs with different morphologies and extracts some generalized principles that can be used as a guide for the design of artificial ones. It starts to develop a toolbox with general tailoring principles for a methodological design of novel soft sensing systems for soft robotics. 

## 3. Hybrid and Multifunctional Units and Systems

Many works in soft robotics look for ways to exploit the mechanisms and material properties, in order to integrate actuation and sensing ability in the same component. Self-sensing actuators have in fact an extensive range of technological implications for improving systems dynamics. In Nature, we see that oriented active fibers are fundamental structures responsible for movements, from the separation of genetic material during cell division, to larger scale movement of muscular structures. With this in mind, the research paper by Ceron et al. [2] shows how to merge soft, inflatable fiber-reinforced actuator functionality with textile fabrication methods, enabling rapid soft actuator fabrication with a diverse range of functional fibers and fabrics. Actuated by inflation, with spiral fiber patterns, the system demonstrates a new approach for the scalable fabrication of synthetic skins that combine local actuation and sensing. 

The use of hybrid systems, with soft-reinforced elements, is not the only approach for actuation and sensing. With respect to various sources, hydrogels have the potential to become a demanding and burgeoning material in soft robotics, in the form of actuators and sensors. As shown in the review from Banerjee et al. [3], the structural properties and mechanics of these components are becoming more and more relevant to soft robotics and especially in biomedical applications. Their hydrophilic nature, swelling/bending capabilities, high biocompatibility, self-healing capabilities, new three-dimensional printing fabrication methods, and stimuli-responsiveness to numerous environmental factors make hydrogels attractive for the improvement of soft robotic systems interaction and mobility.

Moving from single component to a whole-body structure, soft robotics is studying how to better exploit the integration of soft and rigid components and how their balance can be optimized and use the best of their characteristics in a smart way, depending on the application and the environmental context.

Hand exoskeletons, for examples, need to be designed and built around the human hand, conform to the anatomy of the hand itself and be light. Bioinspired soft structures are a champion for their ability to form and adapt to different shapes and complex bodies. The review of the 10-year development of hand exoskeletons for rehabilitation and assistance by Shahid et al. [4], shows a chronological shift toward soft material robotic solutions, and an increase in pneumatic mechanisms versus tendon/cable–pulley ones, which tend to be more lightweight and more conformable to the natural anatomy of the hand that the system has to support.

Slightly differently, when talking of continuum robots to be used as arms for safe item–robot interactions or as a system to move in unstructured environments, the hybrid approach seems to be the more effective one. By integrating rigid and soft components, the system combines versatility, dexterousness and robustness with large bending, rotation and passive stiffness characteristics. The example described by Mishra et al. [5] shows how a light weight, modularity, independent actuation, and compliance can be all optimized and integrated in a smart solution for a soft compliant manipulator.

This combination of adaptability and stiffness variability of soft robotics technologies makes them interesting solutions for biomedical applications. Examples can be found in the review by Varela et al. [6], which discusses how mechanical support strategies for post-myocardial infarction have evolved in recent years. As the adjustability of these devices is highly beneficial, the advent of soft robotics technologies can potentially be very helpful to achieve a more biomimetic mechanical reinforcement. Soft robotics systems can better conform to the heart, perform coordinated movement with the heart wall, and have adaptable mechanical properties over time, such as variable stiffness or anisotropy that can optimize regional mechanical benefit to the infarcted heart.

## 4. Control of Soft Functional Blocks and Systems Autonomy

Soft robotic components and systems have to define new rules for their control, finding new solutions and architectures that can interface with the morphological computation aspects.

The review paper by Mahon et al. [7] is a perspective article on control architecture for soft robots. The paper revised 20 soft systems, with a one-to-one mapping of control outputs to functional blocks, defined as the physical implementation of some concrete behaviors. Stacking functional blocks is the ability to combine functional blocks to obtain more complex system motions and behaviors. However, the story is different for soft robotics, where stacking of functional blocks is seen as a limitative approach. 

More capabilities of the systems will drive them toward practical limits in control, so a higher level of autonomy is required to handle this complexity. In soft robotics, the control cannot be a direct one-to-one mapping, but it will move toward having more functional blocks with fewer control outputs.

## 5. Perspectives for Bioinspired Soft Robotics

The importance of integrating soft, variable-stiffness body parts into robotic systems is the new trend for roboticists who are aiming at increasing the adaptability and robustness of robots in unstructured scenarios, challenging environments, or for a close interaction with humans. 

The approach of soft robotics is becoming more and more popular worldwide, involving a growing multidisciplinary community of researchers. This cross-disciplinary effort and a science-based methodology are needed to advance technologies for soft robotics, where materials science, biology, neuroscience, robotics, and computer science all together are of paramount importance. 

While traditional robots made of rigid links are improving their performance and efficiency in repetitive tasks within structured environments, a new generation of soft robots has the potential to be designed to work outside, in an unstructured external world, as well as within our body.

Looking at how Nature has been able to adapt and survive in dynamically changing environments and in diverse extreme conditions will always be a rich source of inspiration and admirations for us as robotic scientists. Bioinspired robotics and soft robotics are strongly based on this approach, driving the application of robots in a variety of scenarios, including biomedical, service, inspection, search-and-rescue, exploration, and opening new perspectives for improving wellness and quality of life.
